# Absence of photosynthetic state transitions in alien chloroplasts

**DOI:** 10.1007/s00425-019-03187-2

**Published:** 2019-05-27

**Authors:** Anna M. Yeates, Mikhajlo K. Zubko, Alexander V. Ruban

**Affiliations:** 1 School of Biological and Chemical Sciences, Queen Mary University of London, Mile End Road, London, E1 4NS UK; 2 Present Address: Mikrobiologický Institute, Novohradská 237 – Opatovický Mlýn, 37901 Třeboň, Czech Republic; 3 Faculty of Science and Engineering, Manchester Metropolitan University, John Dalton Building, Chester St, Manchester, M1 5GD UK

**Keywords:** Cybrid, LHCII, Non-photochemical quenching, State transitions

## Abstract

**Main conclusion:**

The absence of state transitions in a *Nt*(*Hn*) cybrid is due to a cleavage of the threonine residue from the misprocessed N-terminus of the LHCII polypeptides.

**Abstract:**

The cooperation between the nucleus and chloroplast genomes is essential for plant photosynthetic fitness. The rapid and specific interactions between nucleus-encoded and chloroplast-encoded proteins are under intense investigation with potential for applications in agriculture and renewable energy technology. Here, we present a novel model for photosynthesis research in which alien henbane (*Hyoscyamus niger*) chloroplasts function on the nuclear background of a tobacco (*Nicotiana tabacum*). The result of this coupling is a cytoplasmic hybrid (cybrid) with inhibited state transitions—a mechanism responsible for balancing energy absorption between photosystems. Protein analysis showed differences in the LHCII composition of the cybrid plants. SDS-PAGE analysis revealed a novel banding pattern in the cybrids with at least one additional ‘LHCII’ band compared to the wild-type parental species. Proteomic work suggested that the N-terminus of at least some of the cybrid Lhcb proteins was missing. These findings provide a mechanistic explanation for the lack of state transitions—the N-terminal truncation of the Lhcb proteins in the cybrid included the threonine residue that is phosphorylated/dephosphorylated in order to trigger state transitions and therefore crucial energy balancing mechanism in plants.

**Electronic supplementary material:**

The online version of this article (10.1007/s00425-019-03187-2) contains supplementary material, which is available to authorized users.

## Introduction

The acquisition of chloroplasts derived from free-living, cyanobacteria-like, prokaryotic organisms marked the advent of all photosynthetic eukaryotic life (Schmitz-Linneweber et al. 2005; Archibald 2009). The transformation of endocytosed prokaryote into obligate organelle entailed massive gene transfer from the endosymbiont to the host nucleus (Allen et al. 2011). The relocation of genetic material necessitates that the proteins it encodes are imported back into the organelle, which requires transport across several membranes as well as the cytoplasm, targeting to a specific site and usually, the assembly into large oligomeric complexes (Blankenship 2002). Given the complex logistics that gene relocation imposes, it is remarkable that almost the entire genome has been transferred to the nucleus, and implies that there is a strong evolutionary advantage in such translocation. Despite this, an estimated highly conserved 1–5% of the original endosymbiont genome still remains in the chloroplast (Martin and Herrmann 1998) and maintains the role of encoding, transcribing and translating some of the core proteins of the electron transport chain (Blankenship 2002; Allen et al. 2011). This suggests that there is an even stronger evolutionary advantage of retaining the coding material for these particular proteins in the chloroplast. The alliance and stoichiometry of the nucleus and chloroplast-encoded photosynthetic components is of fundamental importance for plant fitness, perhaps most notably in the relationship between the chloroplast-encoded photosystem reaction centres (RCs; Eberhard et al. 2008) and the nucleus-encoded light-harvesting complexes (LHCs) by which they are served.

The need for tightly coordinated interaction between these components is best observed in the molecular mechanisation against the constant irregular, short-term flux of the photosynthetic light environment (Ruban 2009). This flux can be caused, for example, by the dappling effects of an overhead canopy and changes in cloud cover (Ruban 2009). Higher plants have developed a network of responses to cope with unpredictable changes in light availability, from leaf orientation to chloroplast alignment. At the molecular level in the thylakoid membrane, mechanisms involving the photosystems and LCHIIs are rapid and immediate responses to changes in light on a timescale of seconds to minutes (Ruban 2009). In unsaturated, low light (LL), when rates of light absorption fall below that needed for optimal photosynthesis, photosystem II (PSII) and photosystem I (PSI), due to their different absorption spectra (peaking at 680 nm and 700 nm, respectively), are differentially favoured causing irregular, thus non-optimal, transportation of electrons through the thylakoid membrane. A mechanism known as state transitions serves to equilibrate light absorption between PSI and PSII by adjusting their relative antenna size by inter-photosystem migration of the major trimeric light-harvesting complex II (LHCII). LHCII migration from PSII to PSI (into State II) is activated by phosphorylation of the LHCII apoprotein at the N-terminus whilst dephosphorylation activates migration from PSI to PSII (into State I). The kinase and phosphatase activation has been shown to be controlled by the redox state of the plastoquinone pool (Allen 2009; Ruban and Johnson 2009; Puthiyaveetil et al. 2012).

**Fig. 1:**
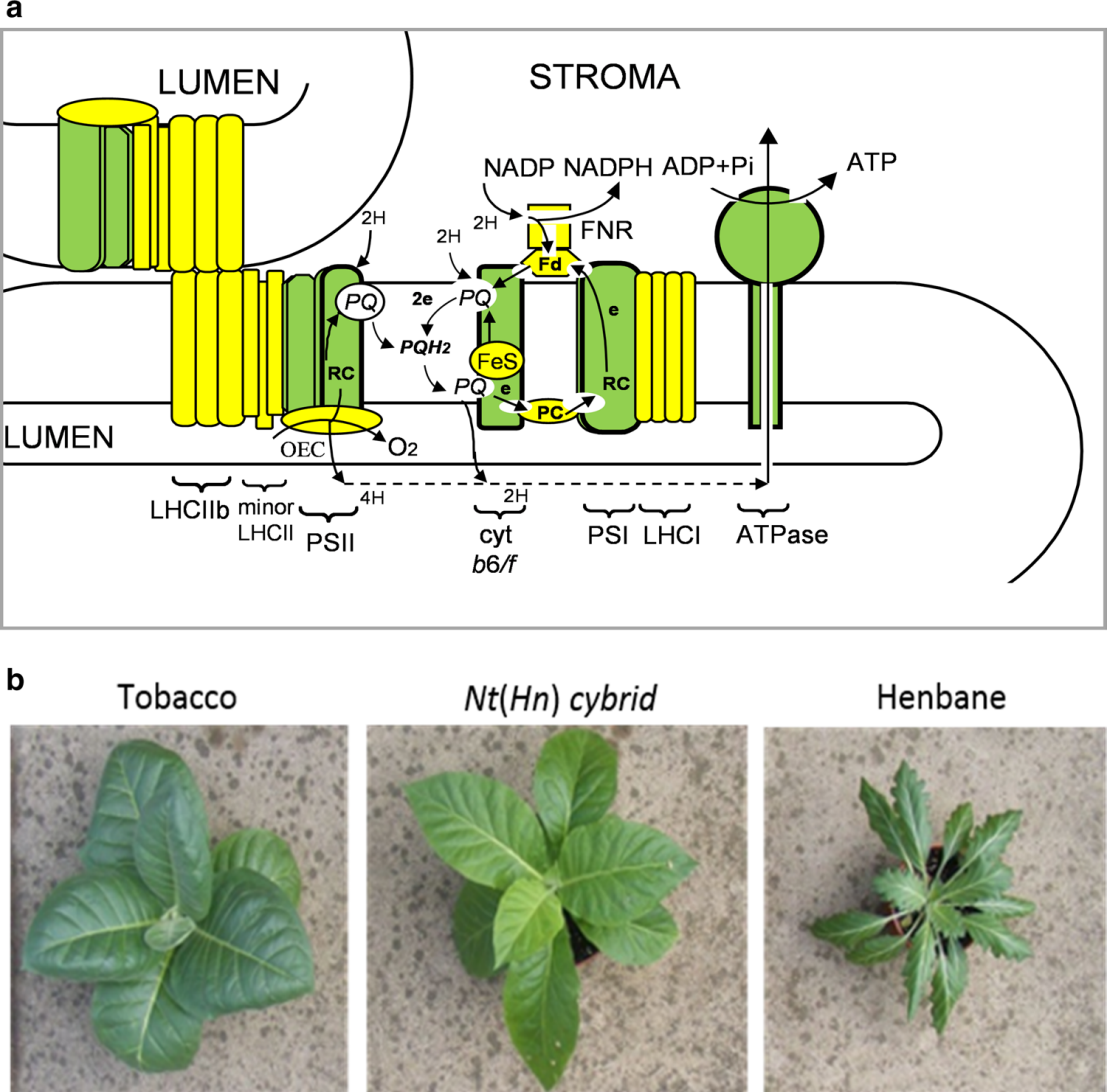
**a** Scheme showing the photosynthetic machinery of the electron transport chain in the thylakoid membrane. Chloroplast-encoded components are in green, nucleus-encoded components are in yellow. **b** Mature plants of tobacco (*Nicotiana tabacum)*, henbane (*Hyoscyamus niger*) and a *Nt*(*Hn*) cybrid. Note the elongated leaves of the cybrid as compared to tobacco

In this study, we investigate the state transitions in a novel model in which alien henbane (*Hyoscyamus niger*) chloroplast-encoded proteins function with tobacco (*Nicotiana tabacum*) nucleus-encoded proteins in vivo, in a stable, cytoplasmic hybrid (cybrid) plant (Zubko et al. 1996; Fig. 1b), referred to as a *N. tabacum* (+*H. niger*) cybrid or just *Nt*(*Hn*) hereafter. Plant cybridisation is a cell-engineered inter-specific organelle exchange to combine the chloroplasts and cytoplasm from one species with the nucleus of another species. As such, for the *Nt*(*Hn*) cybrid plant, the green components in Fig. 1a represent henbane chloroplast-encoded proteins whilst the yellow components represent tobacco nucleus-encoded proteins.

Cybrids have been used in genetic studies towards manipulations in crops soon after the first report on successful somatic hybridisation (Carlson et al. 1972), in particular, after a well -elaborated work on cytoplasmic hybrids (Belliard et al. 1979). One of the advantages of the cybrid model is that it allows to avoid nucleic heterozygosity characteristic of sexual hybrids (Kumar and Cocking 1987; Zubko et al. 2002). It also allows for specific crosses not obtainable by sexual means (Dodds and Roberts 1985; Negrutiu et al. 1989) even between taxonomically distant species (Kushnir et al. 1987). Cybrids therefore offer ongoing potential in plant research and application, for example, in crop agriculture (Dix et al. 2000; Grosser and Cmitter 2004; Liu et al. 2005).

Cybrid studies in photosynthesis research are rare and have never been performed on the new *Nt*(*Hn*) cybrid, unusual in its relative stability and ease of cultivation. We have taken the opportunity to investigate the effects of tobacco–henbane cybridisation on photosynthetic structure and functionality with the aim of further elucidating some of the underpinning mechanisms. In this study, we show the modifications of LHCII in *Nt*(*Hn*) cybrids alongside measurements of state transitions.

## Methods

### Plant material and growth conditions

*Nt*(*Hn*) cybrid plants containing the nuclear genetic material of tobacco and the chloroplast genetic material of henbane were created by one-step protoplast fusion (Zubko et al. 1996). A plant resulting from an independent fusion event, line Rhn3 (Zubko et al. 1996), was then backcrossed with pollen from wild-type tobacco for at least four generations before the seeds were used in this study. Wild-type tobacco (cv. Wisconsin 38) and wild-type henbane (supplied by Oxford Botanical Gardens) were used as controls.

*Nt*(*Hn*) cybrids, tobacco and henbane were grown from seed in Sanyo plant growth cabinets with an 8-h photoperiod at a light intensity of ~ 70 µmol photons m^−2^ s^−1^ and a day/night temperature of 22/15 °C, respectively. After about 4–6 weeks, plants were transferred to growth shelves under 80–100 µmol photons m^−2^ s^−1^. Mature plants were transferred to greenhouses for flowering. For seed production, anthers were removed from flowering *Nt*(*Hn*) cybrids and pollinated with wild-type tobacco pollen. Pollinated flower heads of *Nt*(*Hn*) cybrids, tobacco and henbane were sealed in paper bags to avoid cross-pollination and aid seed collection.

### State transition chlorophyll fluorescence measurements at room temperature

Chlorophyll fluorescence was measured from dark adapted leaves at room temperature using a Dual-PAM 100 chlorophyll fluorescence photosynthesis analyser (Heinz Walz, Germany). Measurements were recorded from attached leaves clamped between LEDs illuminating both the adaxial and abaxial surfaces of the leaf. Superimposition of far-red light (FR; setting 7; Walz) over a weak actinic light (AL, 6 µmol photons m^−2^ s^−1^) in 15 min on/off intervals was used to induce LHC migration between PSI and PSII. The full light programme represented schematically in Supplemental Fig. 1 could be described here briefly. First, a measuring light (ML; 9 µmol photons m^−2^ s^−1^) was applied, and then at 30 s, a SP (saturating pulse; 2000 µmol photons m^−2^ s^−1^) was given. At 2 min, AL + FR were activated. After 15 min, FR light was turned off to induce LHC migration to PSI (into State II), and 15 min later, FR was turned back on to induce a return to State I. The cycle was repeated a second time. SPs (open-headed arrows, Supplemental Fig. 1) were given 30 s after the start and before the end of the FR light application to reveal maximal fluorescence (F′m).

From the acquired traces, three parameters were calculated from several fluorescence transients in order to quantitatively compare the capacity for state transitions in each plant (Ruban and Johnson 2009). They are described schematically in Supplemental Fig. 1. Firstly, a measure of energy imbalance (IB) upon removal of far-red light is defined as (*F*_s_I′ − *F*_s_I)/F_o_; this is primarily a tool for optimising relative light intensities and filters; typically, IB is 0.5–0.7. Secondly, qT, defined as (*F*_m_I − *F*_m_II)/*F*_m_I, reflects differences in the PSII cross section between State I and State II and is variable in the range 0–0.25. Finally, qS, defined as (*F*_s_I′ − *F*_s_II′)/(*F*_s_I′ − *F*_s_II), indicates how full state transitions rebalance energy flow between the photosystems and has a value of 0–1, 1 indicating 100% efficiency (see Supplemental Fig. 1; Ruban and Johnson 2009).

### State transitions measurements by 77 K fluorescence emission spectroscopy

State transitions were induced at RT using the method described above with the exception that a paper stencil was attached to the leaf prior to light application, to exactly demarcate the area of treatment. When the desired point pertaining to STI or STII (marked as ‘I’ or ‘II’ in Supplemental Fig. 1) was reached, the leaf was removed and a leaf disc was cut out from the centre of the treatment area using a cork borer (5 mm diameter, Usbeck) and frozen in LN2. The leaf disc was ground to homogenate with 1 mL of ice-cold buffer containing 10 mM HEPES at pH 7.6. (For STII, the buffer also contained 10 mM of MgCl and 10 mM NaF to maintain the phosphorylation of LHCII.) The sample was injected into a precooled custom-made sample holder and frozen in LN2. Low-temperature (77 K) fluorescence emission spectra (600–800 nm) were recorded using a Jobin–Yvon FluoroMax-3 spectrophotometer equipped with a LN2 cooled cryostat. Excitation was defined at 435 nm with a 5-nm spectral bandwidth. The fluorescence spectral resolution was 1 nm. Spectra were normalised at 685 nm (PSII maximum) in order to observe relative changes in fluorescence from PSI (735 nm).

### Isolation of unstacked thylakoids

Unstacked thylakoid membranes were prepared from dark adapted leaves (1 h) using the following procedure which was performed in the dark with samples on ice where possible: 80 g of fresh leaf material (about four tobacco plants) was cut from plants, and midribs were removed. The material was homogenised to pesto consistency in ice-cold slushy grinding buffer (300 mL; 330 mM sorbitol; 10 mM Na_4_P_2_O_7_; and pH 6.5) using a Polytron blender (Kinematica GmbH, Switzerland). The homogenate was first filtered through two layers of muslin tightly squeezed to force out the suspension and then filtered gently through 2 × 2 layers of muslin, between which was one layer of cotton wool for starch removal. The filtrate was centrifuged (4000 g; 10 min; 4 °C), and the pellet was resuspended in a small volume of washing medium (330 mM sorbitol, 10 mM MES; pH 6.5) and centrifuged again (4000 g; 10 min; 4 °C). The pellet was resuspended a second time in 30 mL of resuspension medium (330 mM sorbitol; 1 mM EDTA; 50 mM HEPES; pH 7.6). Thylakoids were released from the chloroplasts through osmotic shock by adding 50 mL of break medium (10 mM HEPES; pH 7.6) with mixing for 30 s. The reaction was then stopped by the addition of 50 mL of osmoticum medium (50 mL; 660 mM sorbitol; 40 mM MES; pH 6.5). The thylakoids were centrifuged a final time (4000 g; 10 min; 4 °C), and the pellet was resuspended in resuspension medium (as before) to a final concentration of 2 mg Chl mL^−1^ and stored at − 80 °C until needed. Chlorophyll concentration was determined according to the method of (Porra et al. 1989).

### Isoelectric focusing of thylakoid membranes

Non-denaturing IEF was used to separate LHCII components from thylakoid preparations following the procedure of Bassi et al. (1991) with modifications by Ruban et al. (1994), and a few further modifications were made here. Briefly, a gel slurry (100 mL; 6% (w/v) Sephadex G-75 Superfine (GE Healthcare); 2.5% (v/v) Ampholine carrier ampholites (pH 2.5-5; GE Healthcare); 1% (w/v) glycine; 0.06% (w/v) *β*-dodecylmaltoside (*β*-DM)) was added to a flat-bed gel tray, and, under a fan, 37 g was evaporated off over about 3 h. The gel tray was then transferred to the cooling plate (9 °C) of a Multiphor II electrophoresis system (GE Healthcare). Two IEF electrode strips (GE Healthcare) were saturated, one in anode solution (5.3% (v/v) H_3_PO_4_) and one in cathode solution (1 M NaOH), and carefully positioned at the anode and the cathode positions, respectively. A constant power (8 W) was applied for 2 h to mobilise the ampholytes into a pH gradient along the gel.

Unstacked thylakoid preparations (2 mL) with a total Chl concentration of 2.5 mg mL^−1^ were defrosted on ice and then incubated with 1 mL of 5% (w/v) *β*-DM in water (final *β*-DM concentration 1.67%, w/v) for 60 min during which time they were kept on ice and vortexed every 10 min for protein solubilisation. Samples were loaded into the prefocused IEF gel 2 cm from the cathode and run for 17 h (8 W, 9 °C) to draw the proteins to their isoelectric points. Upon completion, the gel was photographed and a micro-pH meter (Mettler Toledo InLab) was used to determine the pH of the gel at 5-mm intervals.

### Separation of LHC polypeptides by SDS-PAGE

Denaturing SDS–PAGE was used to further separate LHCII components from the main green band of the IEF gels. The main green band was scraped from the IEF gel, and proteins were eluted using a PEGG elution column (GE Healthcare) and elution buffer (25 mM HEPES; 0.01% (w/v) *β*-DM; pH 7.6). The ampholytes were then removed by filtering the sample through a PD-10 desalting column (GE Healthcare). The fresh LHCs were then used in SDS–PAGE. In preparation, 10 µL of sample was added to 10 µL of Laemmli buffer (Laemmli 1970) and heated for 10 min at 90 °C, and then centrifuged at 16 800 × *g* for 5 min.

15–18 µL of the supernatant was loaded into a 15% hand-cast polyacrylamide gel. A pre-stained, broad range marker was also loaded (New England Biolabs). Gels were run at 150 V for 15 min after which power was increased to 180 V for about 1 h or until the dye began to run from the bottom of the gel. Gels were stained with Coomassie brilliant blue for 1 h on a shaker. They were then washed in destaining solution (10% (v/v) ethanol; 10% (v/v) acetic acid; in ultrapure H_2_O) overnight and then stored in H_2_O at 4 °C. Gels were scanned (Epson) for digital records and analysis. Density plots were constructed using ImageJ (Schindelin et al. 2012).

### Sample preparation for proteomic investigation of LHCII

The main green LHC band of the tobacco and *Nt*(*Hn*) IEF gels was collected in four 5-mm-wide strips, and the proteins were eluted as described above. For tobacco, these fractions were then mixed in equal volumes. The four *Nt*(*Hn*) bands were numbered I–IV from cathode to anode, respectively, and were kept as separate fractions. The LHC protein fractions were then separated by SDS–PAGE and visualised by Coomassie stain (as described above). Bands were selected for proteomic investigation, excised using a new scalpel blade, stored individually in eppendorfs with 50 µL of ultrapure H_2_O and sent to Cambridge Centre for Proteomics (CPP; Department of Biochemistry, University of Cambridge, UK; http://proteomics.bio.cam.ac.uk/) for proteomic analysis.

### Proteomic investigation of LHCII

Samples were treated according to CCP’s protocol. In brief, gel bands were destained, reduced with dithiothreitol and alkylated with iodoacetamide, and then digested with trypsin overnight. The supernatant was subjected to automated analysis by liquid chromatography (LC) coupled with tandem mass spectrometry (MS/MS) performed using a nanoAcquity UPLC system (Waters Corp, MA, USA) and an LTQ Orbitrap Velos hybrid ion trap mass spectrometer (Thermo Scientific, Waltham, MA). MS/MS data were converted to.mgf files and then matched against NCBI and UniProt databases using the Mascot search engine (Matrix Science, London, UK) with the following parameters: digestion enzyme = trypsin; maximum missed cleavages = 2; fixed modifications = carbamidomethyl (C); variable modifications = oxidation (M); peptide; mass tolerance = 25 ppm; fragment mass tolerance = 0.8 Da/peptide identifications were accepted if they could be established at greater than 95.0% probability.

### Theoretical calculation of LHC pI and MW upon N-terminal amino acid removal

Tobacco LHC sequences were copied from the UniProt database into the online ExPASy Compute pI tool (Swiss Institute of Bioinformatics, http://web.expasy.org/computepi/). Transit peptides, identified in UniProt, were removed from the sequence. Molecular weight (MW) and the theoretical isoelectric point (pI) of the peptides were calculated using the ExPASy software as the N-terminal amino acid (aa) residues were manually deleted one by one sequentially. Changes in MW (ΔMW) and in pI (ΔpI) were calculated from the MW and theoretical pI of the full, mature, peptide sequence.

## Results

### *Nt*(*Hn*) cybrid plants

*Nt*(*Hn*) cybrid seeds showed delayed germination by ~ 3–5 days compared to the parent plants (tobacco, 8, 9 days; henbane, 7, 8 days) and a low germination rate of 40–50%, compared to 80–90% in tobacco and 70–90% in henbane. These findings were consistent with previous observations (Zubko et al. 1996). On the whole, the *Nt*(*Hn*) phenotype matched that of tobacco (Fig. 1b) with slightly elongated leaves and thinner leaf tissue. Occasionally, abnormal plants appeared with particularly elongated leaves or curling of the leaf edges and stunted growth, these ‘anomalies’ were not used in experiments.

### State transitions were strongly inhibited in *Nt*(*Hn*) cybrids

Chlorophyll fluorescence was measured during the application of a standard light regime for inducing state transitions, details of which are given in Supplemental Fig. 1a. The main principle behind the established protocol for room-temperature state transition measurements using the Chl *a* fluorescence signal, is the selective modulation of PSI electron transport by the addition or removal of far-red light (FR; 730 nm), respectively, whilst PSII is driven continuously by a weak actinic light (AL; 635 nm). In theory, superimposing FR over weak AL preferentially drives PSI compared to PSII, producing an oxidised PQ pool. In turn, the removal of FR inhibits PSI resulting in PQ pool reduction.

State transition measurements were quantified using parameters calculated from measured fluorescence transients (see Supplemental Fig. 1; Ruban and Johnson 2009, for details). Maximal fluorescence (*F*_m_) and minimal fluorescence (*F*_o_) were first measured from dark adapted leaves; then, after ~ 8 min of light induction (AL + FR), two ~ 8-min FR on/off cycles were applied. Measurements were made from the second cycle to avoid the confounding effects of induction kinetics after dark adaptation.

**Fig. 2:**
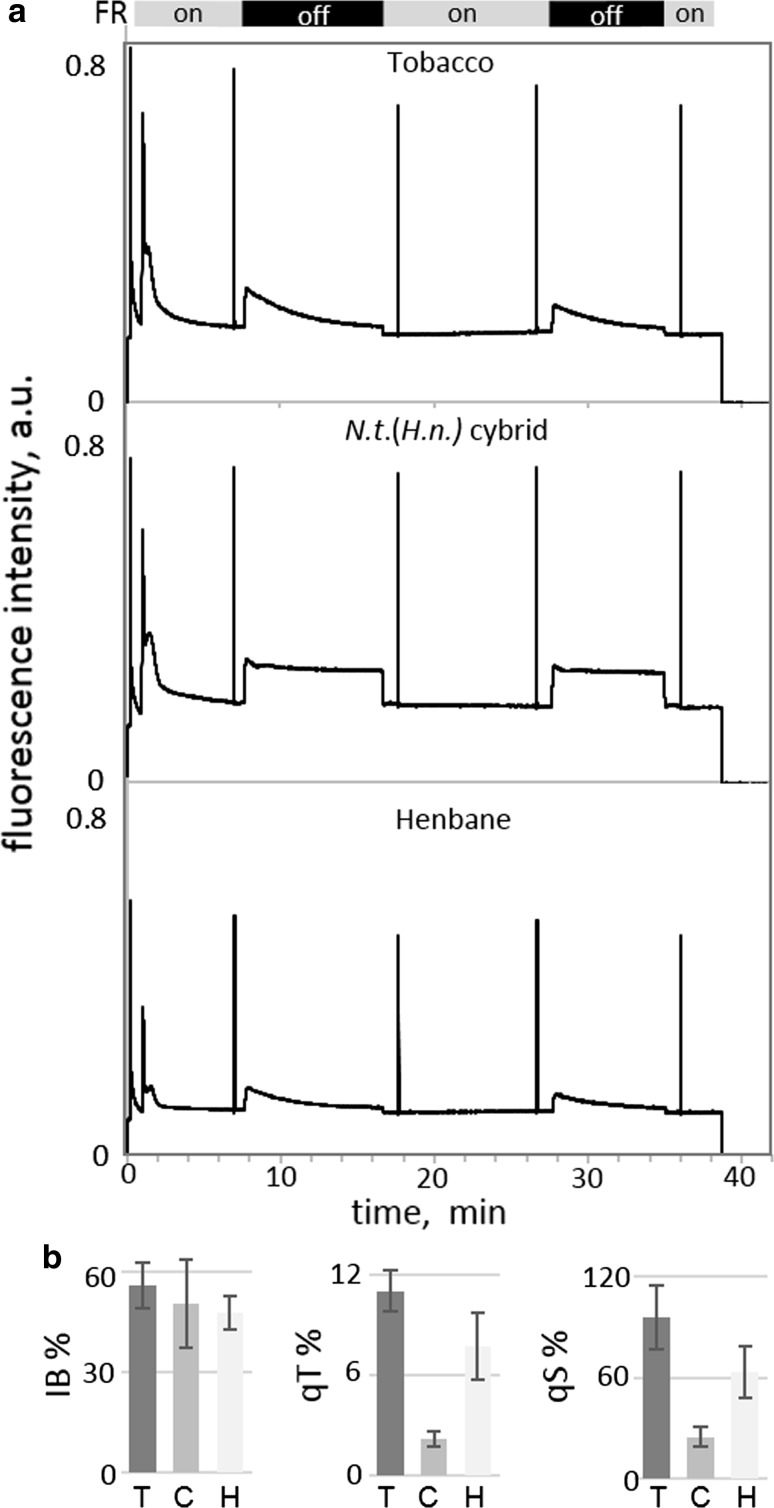
Representative fluorescence traces measured during a PAM state transition induction protocol. **a** Top—tobacco, middle—*Nt*(*Hn*) cybrid and bottom—henbane. **b** State transition parameters, IB, qT and qS. Data are mean average ± standard deviation, *n* > 3. The calculated parameters and the points at which measured parameters were taken are indicated in Supplemental Fig. 1

Representative fluorescence traces from mature tobacco, henbane and *Nt*(*Hn*) are shown in Fig. 2a. In general, the *Nt*(*Hn*) cybrid fluorescence levels at *F*_o_ and *F*_m_ were similar to tobacco rather than henbane. After far-red light was turned off after about 8 min, reducing excitation of PSI, the initial response of the PSII fluorescence level in all three plants was comparable, in that the fluorescence level increased by about 50% of the level of *F*_o_. This can be represented by the imbalance parameter (IB) which was calculated as 56 ± 7% in tobacco, 48 ± 5% in henbane and 56 ± 13% in *Nt*(*Hn*) (Fig. 2b). IB indicates the relative extent of the reduction of the PQ pool and thus indicates the signal strength for activation of the kinase involved in phosphorylation of PSII.

After the sharp fluorescence increase following FR removal at ~ 8 min, the subsequent fluorescence signal of *Nt*(*Hn*) plants was markedly different from the parental wild types. In tobacco and henbane, the fluorescence signal decreased quickly, then more slowly as LHCII left PSII and docked at PSI, which reduced the absorption cross section of PSII whilst increasing absorption at PSI, until a steady state was reached near FsI levels (see Supplemental Fig. 1) indicating transition from State I to State II after about ~ 8 min. In contrast, the drop in the florescence signal from *Nt*(*Hn*) was minimal with levels remaining closer to *F*_s_I′. The extent of the return to F_s_ levels is designated as qS and reflects the ability to rebalance electron flow across the thylakoid membrane; qS was calculated as 96 ± 19% and 63 ± 15% in tobacco and henbane, respectively (where 100 indicates complete efficiency; Ruban and Johnson 2009). qS in the *Nt*(*Hn*) cybrid was comparatively very low at 24 ± 6% (Fig. 2b).

Additionally, henbane and tobacco showed the expected reduction in maximal fluorescence (in the light) from *F*_m_I′ (in State I) to *F*_m_II′ (in State II), whereas little or no change was seen in the level of maximal fluorescence between *F*_m_I′ and *F*_m_II′ in the cybrid (see Supplemental Fig. 1 for parameter descriptions). The change in maximal fluorescence is expressed as the parameter qT and reflects the change in PSII cross section with a range from 0 to 0.25 (Ruban and Johnson 2009). Values of qT in tobacco, henbane and *Nt*(*Hn*) were calculated as 11.0 ± 1.2%, 7.6 ± 2.0% and 2.2 ± 0.4%, respectively (Fig. 2). A low qT value in the *Nt*(*Hn*) cybrid suggests that LHCII did not separate from PSII to join PSI. A return to *F*_m_′ levels in the wild-type plants after a further 8 min of far-red light treatment indicates that the process was reversible and not due to photoinhibition.

Additional confirmation of the absence of the state transitions in the cybrid was obtained by using the 77 K fluorescence measurements on isolated chloroplasts. Supplemental Fig. 2 shows that whilst in the tobacco and henbane the relative contribution of the 735-nm band corresponding to PSI was increased in State II, there was no detectable increase in this band for the cybrid. This indicates that there was neither LHCII detachment from PSII complex nor its attachment to PSI (Ruban and Johnson 2009).

### Separation of *Nt*(*Hn*) cybrid LHCII complexes by IEF

**Fig. 3:**
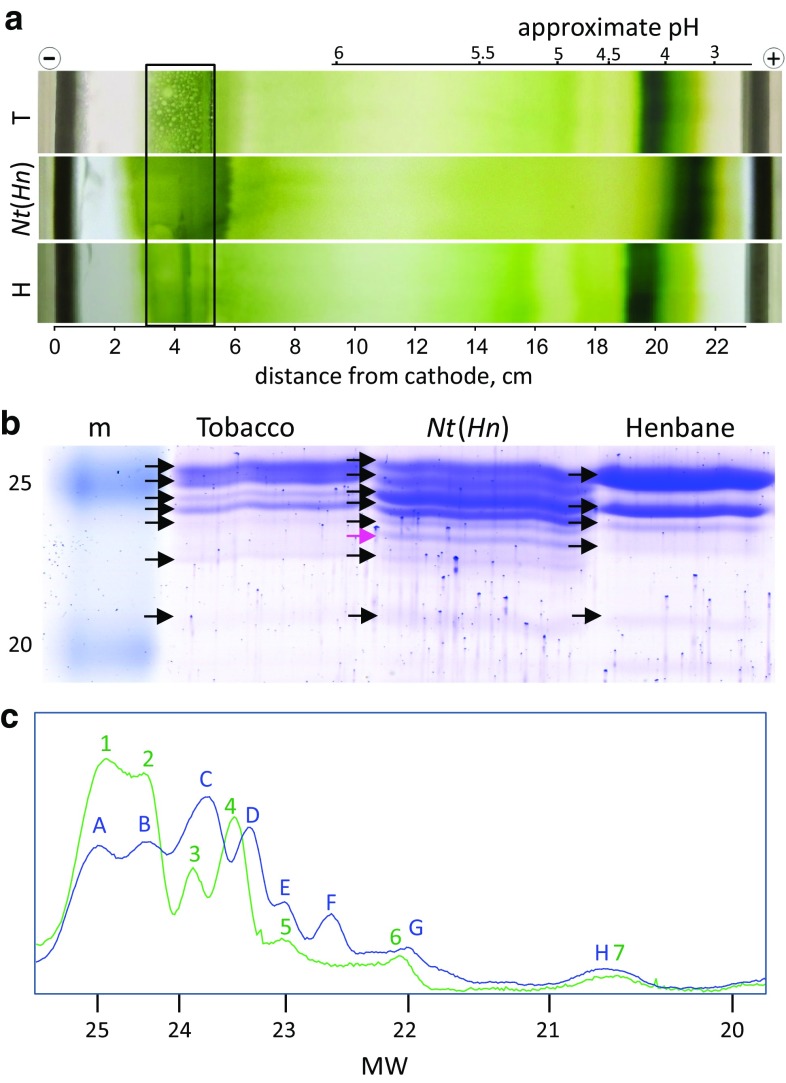
Isolation of light-harvesting complexes. **a** Thylakoid protein preparations separated on the basis of pI by isoelectric focusing. Photographs of representative gels from tobacco (T), the *Nt*(*Hn*) cybrid and henbane (H). The boxed area near the cathode defines the site where thylakoids were loaded into the gel. The pH scale and distance from cathode are marked above and below the gel, respectively. The former is approximate as pH gradients varied slightly between gels. Images have been cropped to show the central aspect of the gel tray for easy comparison. **b** SDS–PAGE separation of LHCII polypeptides from the broad IEF band in a 15% acrylamide gel. The protein marker is shown in the first lane (m) the standard weights are indicated. The pink arrow indicates the novel band in the *Nt*(*Hn*) separation. **c** Density plots of the MW profiles shown in **b**. The plots from left to right correspond to the top to bottom of the gel in **b**, respectively. Tobacco is shown in green and the *Nt*(*Hn*) cybrid in blue. Peaks were numbered 1–7 in tobacco with equivalent peaks in *Nt*(*Hn*) density plots assigned letters (*A*–*H*)

In order to understand the cause of inhibited state transitions in the cybrid, we first undertook the isolation of light-harvesting antenna complexes of PSII. Non-denaturing IEF was employed to separate the major LHCII from thylakoid preparations of wild-type tobacco, henbane and *Nt*(*Hn*) cybrids (Fig. 3a). In all cases, a densely pigmented LHCII band was resolved, with, a clearly, a very different migration pattern in the *Nt*(*Hn*) cybrid compared to either tobacco or henbane (Fig. 3a). Indeed, whilst the tobacco and henbane LHCII focussed at about pH 4.0 and 4.2, respectively, the hybrid LHCII migrated to the pH region of around 3.5 (Fig. 3a). Bands at around pH 5.0 and above were originated from various PSII and PSI core complexes as described in (Dainese et al. 1990; Ruban et al. 1994).

### Further separation of LHCII complexes by SDS–PAGE reveals novel banding patterns

To further resolve the LHC content of the IEF separation, the broad green band was collected and the proteins were analysed by SDS–PAGE (Fig. 3b). In the region of 25 to 20 kDa, a total of seven clear bands were counted in the tobacco sample and five in the henbane sample (Fig. 3b). *Nt*(*Hn*) LHC separation revealed eight bands, demonstrating the presence of at least one novel band as compared to tobacco, suggested to be the band indicated by the black arrow (Fig. 3b). Other differences in MWs of the band products and stoichiometry between the *Nt*(*Hn*) cybrid and its nuclear parent were also apparent.

Densities of bands in tobacco and the *Nt*(*Hn*) cybrid were measured and plotted to aid comparative analysis (Fig. 3c). The peaks of the seven tobacco bands are numbered 1–7 whilst the eight cybrid bands are indicated by letters (*A*–*H*; Fig. 3c). For henbane, only six bands were clearly present with bands 1 and 2 of tobacco forming one broad band in henbane, and band 3 is seemingly not present at all (data not shown) demonstrating that the LHC proteins in the cybrid were of tobacco origin.

The banding in *Nt*(*Hn*) differed from tobacco in two ways: firstly, in the number and alignment of the bands and secondly, in protein stoichiometry. In the first case, *Nt*(*Hn*) peaks A, B, E, G and H aligned well to the wild-type bands 1, 2, 5, 6 and 7, respectively. Bands, C and D, were shifted to slightly lower MW with respect to the equivalent bands 3 and 4 in tobacco. However, the apparent MW change may be an artefact of relatively high protein content of peaks C and D, causing the bands to spread into a lower MW region. Interestingly, band F in *Nt*(*Hn*) was novel, without an equivalent in the tobacco or henbane polypeptide separations (Fig. 3b, c). It had a fairly large relative protein content at 6.7% of the total protein content (Table 1) and was clearly defined. A possible hypothesis to explain both the appearance of the novel band F and the reduced protein content of band A and B relative to tobacco is that band F proteins might be a subset of truncated forms of A/B band proteins.

**Table 1:** MW (kDa) and percentage content of total protein area of the LHC bands shown in Fig. 3c

T bands	1	2	3	4	5	6	7
MW	25.1	24.6	23.9	23.6	23.1	22.2	20.7
SD	± 0.30	± 0.03	± 0.04	± 0.10	± 0.10	± 0.21	± 0.63
%	35.1	20.6	9.4	17.8	7.3	4.5	5.2

*Nt*(*Hn*) bands	*A*	*B*	*C*	*D*	*E*	*F*	*G*	*H*
MW	25.0	24.7	23.7	23.3	22.9	22.6	21.9	20.6
SD	± 0.14	± 0.21	± 0.12	± 0.15	± 0.11	± 0.23	± 0.22	± 0.31
%	20.4	17.2	25.4	13.7	6.1	6.7	7.5	3.0
ΔMW	2.4	2.1	1.1	0.7	1.3	–	–	–

The bottom row shows the difference (Δ) in MW (kDa) between each *Nt*(*Hn*) cybrid band and band *F*. Data are mean average ± SD, *n* = 3

To aid the identification of the SDS–PAGE components, calculations of theoretical pI and MW of mature tobacco minor and major PSII extrinsic antennas were made from published sequences that were available in UniProt, using ExPASy software (Artimo et al. 2012). Calculations and UniProt accession numbers are presented in Table 2.

**Table 2:** Theoretical pI and MW of LHC antenna proteins of tobacco

Protein	UniProt acc no.	MW (kDa)	pl
lhcb 1.1	P27496	24.926	5.40
lhcb 1.2	P27493.1	24.707	5.01
lhcb 1.3	P27495.1	24.870	5.13
lhcb 1.4	P27491	24.908	5.10
lhcb 1.5	P27492	24.882	5.13
lhcb 2.1	P27494.1	24.784	5.02
lhcb 3.1*	A0A076L1Y1	24.216	4.84
CP29 (lhcb 4)	Q0PWS7	28.469	5.46
CP26 (lhcb 5)	Q0PWS5	23.962	5.00
CP24 (lhcb 6)	Q0PWS6	22.880	4.96
PsbS	Q9SMB4	21.929	4.70

Calculations were made using the sequences available in UniProt database together with the ExPaSy compute pI/MW tool

*For pI/MW calculation from mature peptide, the transit peptide indicated by Babiychuk et al. (1995) was removed manually from the available sequence

According to the literature (Aro et al. 2005; Caffarri et al. 2009), and to predicted MWs (Table 2), other than the major (lhcb1-3) and minor (CP24, CP26 and CP29) antenna proteins (polypeptides), the proteins comprising other bands, particularly at low MW, are likely to be PsbP (OEC23), PsbQ (OEC16) and PsbS. Interestingly, peak 3 (not detected in henbane) had a comparatively low yield at 9.4% in tobacco (calculated from the total area of all seven tobacco bands), whereas the equivalent peak C in the *Nt*(*Hn*) profiles had a relatively large percentage content (25.4%).

### Proteomic analysis of LHCII proteins

To test for N-terminal truncation, the most definitive approach would be to sequence the N-terminus. However, all N-terminal sequencing techniques require protein purity, which is considered unobtainable in natural samples of LHCII due to the similarity of the closely related monomers of lhcb1, lhcb2 and lhcb3. Moreover, lhcb1 is known to have at least five isoforms (lhcb 1.1–1.5) with some estimates of more (see Table 2). We therefore conducted proteomic analysis of the LHCs by tandem mass spectrometry (MS/MS). This method would not allow to confirm an absence of the N-terminus in the *Nt*(*Hn*) cybrid because an absence in the data might only be the result of a failure to recover the peptide sequence during MS/MS. However, if the N-terminus was detected, this would confirm its presence.

For mass spectrometry, we newly collected the main green band from IEF separations of tobacco and *Nt*(*Hn*) thylakoids in four 0.5-mm-wide strips. For the *Nt*(*Hn*) cybrid, these fractions were differentiated from cathode to anode as I–IV and pIs were 4.1–3.8 (I), 3.8–3.5 (II), 3.5–3.2 (III) and 3.2–2.9 (IV). For tobacco, the four fractions were mixed together in equal quantities. The reason for fractionating the IEF band was firstly to gain a better resolution of the protein pI based on the distribution within the main LHCII band, and secondly, to enable selection of perhaps purer proteins after separation by SDS–PAGE (Supplemental Fig. 3).

Bands in the SDS–PAGE gel were identified as previously described in Fig. 3b, c. The extrapolation of the *Nt*(*Hn*) band into four fractions, across four lanes, revealed a general trend for lower MW bands to appear in more acidic IEF fractions. The bands selected for proteomic work were 1 and 2 from tobacco, estimated to contain the LHCII proteins, and *A*–*F* for *Nt*(*Hn*), from which the principle bands of interest were C and D, due to their increased protein content, and the novel band F. The selection of the bands, indicated in Supplemental Fig. 3, was based on high concentration and clear resolution in the gel.

Retrieved peptide sequences were searched against UniProt and NCBI databases. The results of the Mascot search are summarised in Supplementary Table 1. The top five hits are given for each band. Where alignments were found against *Nicotiana sylvestris* proteins, we used BLAST for searching the *N. sylvestris* sequence against tobacco in the Solgenomics database (http://solgenomics.net), which has a more up-to-date and comprehensive genomic data set for the Solanaceae family. In all cases, we obtained 98–100% sequence alignment with tobacco. Therefore, *N. sylvestris* alignments may be considered equivalent to alignments against tobacco. The sequence similarity is because *N. sylvestris* is the maternal progenitor of tobacco and the male progenitor is *N. tomentosiformis* (Hasegawa et al. 2002; Yukawa et al. 2005).

Strong sequence similarity (%) was found between the MS-determined peptide sequences and the matched database sequences in all cases, for at least the top five hits (Supplementary Table 1). This suggests that there was contamination of proteins across the excised bands. At least two isoforms of lhcb1 were found in every sample. Lhcb2.1 was also matched to peptides from several bands. The minor antennas, CP26 and CP29, were present in both tobacco bands and in band B of *Nt*(*Hn*).

Matched protein sequences are presented in Table 3. We show amino acids starting from the 27th residue of the transit peptide at the N-terminus to the 60th residue which resides within the mature protein sequence. Although alignments were made only from mature proteins, the transit peptide sequence is included for reference and is highlighted on a black background. The start of the mature polypeptide usually begins one residue downstream from the leading methionine (Michel et al. 1991). Peptide alignments determined by MS/MS are highlighted in grey. N-terminal threonine residues of the lhcb1 and lhcb2 proteins are highlighted in red (Table 3). These are the sites of phosphorylation that triggers the detachment of LHCII from PSII for migration to PSI in state transitions.

**Table 3:**
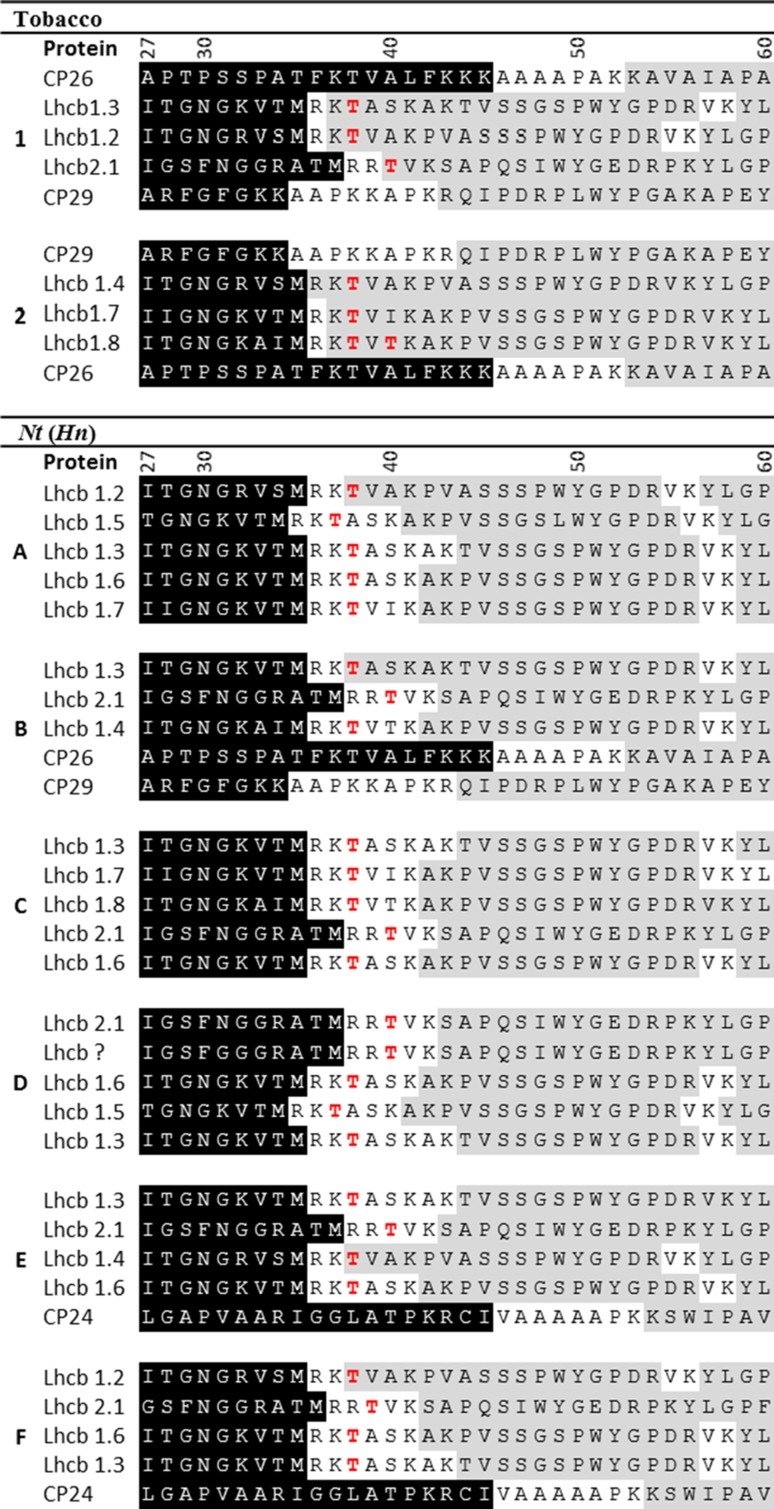
Summary of Mascot search results

MS/MS data were searched against NCBI and UniProt databases for protein identification, using the Mascot search engine. The top five ‘hits’ are listed for tobacco samples 1-2, and *Nt*(*Hn*) samples *A*–*F*. N-terminal sequences are shown from residues 27–60. The first 26 residues are removed for clarity, and the remaining transit peptide is shown on the black background. The grey background indicates where peptides identified from MS/MS matched the protein sequence. Phosphorylation sites that are involved in state transitions are shown in red

In order to gauge how the removal of N-terminal amino acids affects the pI point and MW of LHC proteins, mature peptide sequences of lhcb1 isoforms and lhcb2 (UniProt accession numbers in Table 2) were loaded into ExPAsY MW/pI calculation software and MW, and pIs were determined after sequential removal of individual N-terminal amino acid residues. The change in MW (ΔMW) and pI (ΔpI) was determined according to the difference with the full-length mature protein.

**Fig. 4:**
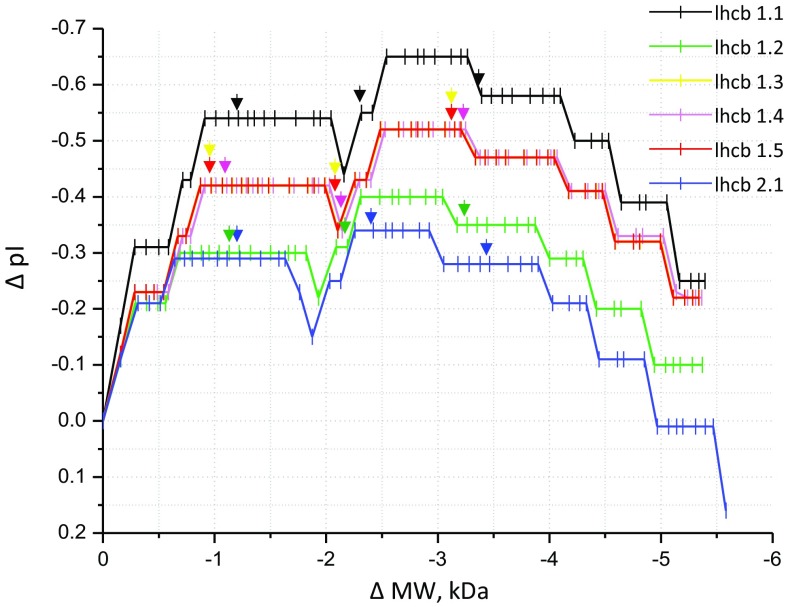
Theoretical changes in pI as N-terminal amino acids are removed. Using ExPAsY MW/pI calculation software, we calculated the pI point and MW of each of the lhcb1 isoforms (lhcb1.1–lhcb 1.6), and lhcb2.1 as amino acid residues were removed one by one. From these data, we calculated ΔpI and ΔpH in respect of the full-length sequence of the mature protein. Each small vertical line in the plot represents the amino acid removal, arrows indicate the tenth, 20th and 30th residue in each polypeptide

Figure 4 shows the plots of ΔpI against ΔMW, calculated for lhcb1 and lhcb2 monomers. The plots show that even the removal of one amino acid, in all cases an arginine, causes a large ΔpI from − 0.21 to − 0.31. The removal of 4–6 amino acids causes a ΔpI of − 0.28 to − 0.43 depending on which lhcb protein has been altered. In lhcb1.1, 1.4 and 1.5, the removal of a further three residues again further reduces the protein pI. The threonine phosphorylation site that is involved in state transitions is the third residue of the mature peptide. In Table 3, peptide alignments at the N-terminus are missing for 6–8 residues in all the matched sequences, with the exception of lhcb1.2 in band A and lhcb1.3 in band B. By comparison in the peptide alignments for the proteins in bands 1 and 2 of tobacco, there are six hits against lhcb1/lhcb2 proteins, all of which include a peptide alignment at the N-terminus, including the threonine residue at position three. We need to emphasise that an unaligned region in mass spectrometry data does not confirm an absence; however, the consistent non-alignment at the N-terminus, seen in all bands of the *Nt*(*Hn*) LHCs (except in bands A and B, where MW was the same as in wild-type tobacco), strongly suggests that the N-terminus of the lhcb1 and 2 proteins was truncated. This is most likely due to misidentification of the transit peptide during cleavage.

## Discussion

In this work, we present evidence that indicates the deletion of the N-terminus of the tobacco lhcb1/2 protein in *Nt*(*Hn*) and we propose that the removal of the threonine at the N-terminus was responsible for the near total inhibition of state transitions.

### A new model for photosynthesis

We have introduced the *Nt*(*Hn*) cybrid plant as a new model for photosynthesis research in which alien henbane chloroplast-encoded proteins function with tobacco nuclear-encoded proteins. We show that the cybrid contains a novel LHCII polypeptide composition. We also present, for the first time, state transition measurements in cybrids, revealing that they are strongly inhibited. The *Nt*(*Hn*) cybrid is an important and exciting model for photosynthesis research for studies of interactions between plant genomes (compartmental compatibility/incompatibility) and for the investigation of the closely coordinated interaction between nucleus- and chloroplast-encoded proteins, in particular those which require specific and rapid integrated mechanisation in response to a rapid flux in the light environment.

### Modification of LHCII composition in cybrids suggests compartmental incompatibility

LHCII polypeptides, key components in photosynthesis, are shown to be altered in the *Nt*(*Hn*) cybrid. To date, only a few studies have investigated cybrid photosynthetic protein composition (Kushnir et al. 1987; Babiychuk et al. 1995; Peter et al. 1999). Kushnir et al. (1987) and Peter et al. (1999) found that cybrid plants *Nt*(*Ab*) with a tobacco nuclear background and plastome of deadly nightshade (*Atropa belladonna*) had altered expression of membrane proteins in the 27–25 kDa weight range. A later work by Babiychuk et al. (1995) showed similar results in five new cybrid combinations, all of which combined a tobacco nucleus with the plastome of different species from the *Solanaceae* family.

Immunoblots of additional polypeptides of novel 26, 24.5 and 24 kDa bands in *Nt*(*Ab*) cybrids were successful against anti-LHCII polyclonal antibodies showing that the polypeptides were in fact derived from LHCII nucleus-encoded genes (Babiychuk et al. 1995). Sequencing analysis of a novel 26 kDa polypeptide band in a cybrid of *Nicotiana plumbaginifolia* with an *A. belladonna* plastome showed that it aligned with wild-type lhcb1 polypeptide revealing a 11 and 12 amino acid truncation at the N-terminus (Babiychuk et al. 1995).

The regularity of LHCII alterations in these cybrids implies a shared compartmental incompatibility syndrome of the plastome to the nucleus. The consistency of these findings throughout these cybrids may correlate with the fact that the cybrid LHCII proteins analysed so far were made for cybrids in which the nuclear parent was tobacco, with the one exception in which it was *N. plumbaginifolia*, and the plastome parent was another species of the Solanaceae family. Perhaps this case indicates an incompatibility peculiar to the inter-familial combination. LHCII protein analysis in a Solanaceae cybrid of a Peruvian nightshade (*Lycopersicon peruvianum*) nuclear parent and tomato (*Lycopersicon esculentum*) plastome parent showed no difference in LHCII polypeptide composition (Kochevenko et al. 2000). This inter-specific nucleo-plastome combination seems to be photosynthetically more compatible than the inter-generic combinations. The differences found to occur between cybrids, in addition to the observed incompatibility in cybrids in general, suggest that plant nuclear and chloroplast genomes reflect species-specific co-evolution.

### N-terminal truncation was the cause of inhibited state transitions in *Nt*(*Hn*) cybrids

Given the reports on LHC alteration in various cybrid plants, and furthermore that the reports of alterations are all based on cybrids with a tobacco nuclear parent, it seems reasonable to suggest that similar changes occurred for LHCII proteins of *Nt*(*Hn*).

Based on the discovery, during IEF, that *Nt*(*Hn*) LHCs had novel, lower pIs, we investigated *Nt*(*Hn*) LHCII proteins for modification, with the expectation that the analysis might provide an explanation for state transitions inhibition in *Nt*(*Hn*). MW-based separation of tobacco LHCs collected from IEF gels showed seven bands. Seven bands following the same basic migration pattern were also found in the *Nt*(*Hn*) cybrid. An additional eighth band (band F; Fig. 3b, c) in *Nt*(*Hn*) was novel in that it was not present in either of the parent species. Proteomic analysis of each *Nt*(*Hn*) sample identified protein content as, mostly, various isoforms of lhcb1/2 proteins from tobacco. From each of bands A, B, E and F, a single match was made against an lhcb1 isoform with almost-complete N-terminal sequence coverage; in all other matches against lhcb1/2 isoforms, the sequence coverage did not include the first 5-8 N-terminal residues. In contrast, all tobacco peptide sequences showed coverage at the N-terminus with the exception of only the first 1–2 amino acids. The results suggest that the N-terminus had been lost from a significant subset of the *Nt*(*Hn*) lhcb polypeptides.

The findings were somewhat surprising in that although, based on previous cybrid research, we expected that N-terminal removal might be the cause of novel pI and MW, we expected to find the truncated polypeptides in the novel MW band, namely the band F, and perhaps as additional co-migratory proteins, in the other relatively low MW bands (e.g., bands *C*–*E*). However, peptide coverage of the N-terminus was absent to a comparable degree throughout the results for all the bands, including the relatively high MW bands A and B. The five proteins (with highest sequence coverage) identified in each of the six *Nt*(*Hn*) samples were mostly of the same few types, namely lhcb1/2 isoforms, which have almost identical MW (Supplementary Table 1) and (based on the proteomic data) similar sequence coverage throughout (Table 3). It contradicts the notion that they migrated to six different, distinct positions in the gel. The results instead support the hypothesis that the excised gel bands were all subject to protein contamination from the same source(s), probably just one or two of the high protein concentration bands. It is most likely that contamination occurred during gel band excision.

If the results presented in Table 3 represent contaminant proteins, then they are not indicative of the bulk protein content of the bands, and the real identity of the proteins in the bands remains unknown. Nevertheless, the data do indicate that a subset of lhcb1/2 proteins in which the N-terminus was truncated, was present in the *Nt*(*Hn*) cybrid and furthermore that the cleaved terminus included the threonine that is the site of phosphorylation in state transitions.

Based on our isolation of LHCII from thylakoid preparations, as well as on measurements of photosynthetic function and protein quantification, we know that the truncated lhcb proteins were present in the thylakoid membranes of the *Nt*(*Hn*) cybrid, in comparable quantities to those of the wild-type parental species. Furthermore, grana of comparable size and number to the parental species were observed in TEM micrographs of *Nt*(*Hn*) sectioned leaf tissue (see Supplemental Fig. 4). The majority of LHCII proteins are located within the granal stacks (van Amerongen and Croce 2007), and the stacking of thylakoids into grana has been shown to depend upon the presence of LHCII (Standfuss et al. 2005). The N-terminal cleavage therefore occurred either after the LHC protein (LHCP) arrived at its final destination in the thylakoid membrane or, if it occurred sometime prior to delivery, the truncation of the LHCP did not notably affect the delivery pathway.

State transitions are triggered by phosphorylation and dephosphorylation of the threonine residue located at the N-terminus (Forsberg and Allen 2001) in lhcb1 and lhcb2. If the N-terminus in the *Nt*(*Hn*) cybrid was cleaved above the site for phosphorylation, then it would not be surprising that state transitions were strongly impeded given the removal of the state transitions activation site. Indeed, the results presented here show not only that LHCII in *Nt*(*Hn*) does not dock to PSI but that it does not leave PSII, suggesting that the action necessary for dissociation of LHCII from PSII is impeded possibly by the removal of the activation site, giving further evidence to the theory that the threonine phosphorylation site is the activation site for state transitions.

### Possible points of lchb mis-processing

The point of mis-processing of the tobacco nuclear-encoded lhcb protein in the *Nt*(*Hn*) cybrid presumably involves a henbane chloroplast-encoded factor. The transport of nuclear-encoded chloroplast proteins to their final location within the chloroplast involves the passage across several compartments and membranes, including import into the chloroplast through the TOC–TIC membrane gateway (Chen et al. 2018; Cline and Henry 1996; Fuks and Schnell 1997; Paila et al. 2015; Soll and Schleiff 2004) and cleavage of the N-terminal chloroplast transit peptide (cTP) by a general stromal processing peptidase (SPP; Chotewutmontri et al. 2017; Oblong and Lamppa 1992; Richter et al. 2008). Following this, the mature lhcb protein translocates to the thylakoid via the chloroplast signal recognition particle (cpSRP) pathway (Gao et al. 2015). cpSRP interacts with a thylakoid membrane-bound receptor, and the mLHCP is inserted into the membrane by an insertase.

As yet, no chloroplast-encoded factor has been identified in the pathway of the lhcb protein from cytosol to its position on the thylakoid membrane and the only known cleaving mechanism in the process is of the cTP by SPP. However, some aspects are not fully understood (Bouchnak et al. 2019; Gao et al. 2015; Stengel et al. 2008) and could involve an, as yet unidentified, chloroplast-encoded factor. In the plastid protease system, only a single chloroplast-encoded component, the proteolytic ClpP1 (caseinolytic protease P1) subunit, has been identified (Adam et al. 2001; Nishimura et al. 2016; Kuroda and Maliga 2003). The Clp protease complex (Adam et al. 2001) comprises several other subunits all of which are nuclear encoded. In plastids, Clp is located in the stroma and is responsible for the degradation of both soluble and membrane-bound proteins (Adam 2000; Adam et al. 2001) although there is no direct evidence that LHC proteins are a substrate for Clp.

## Conclusion

Cytoplasmic hybrid plants of *Nicotiana tabacum* endowed with *Hyoscyamus niger* chloroplasts, *Nt*(*Hn*), show novel and interesting alterations in LHCII manifested mechanistically in unusual state transitions which are severely inhibited. The results are considered to be due to a possible truncation of the LHCII N-terminus, which has removed the threonine activation site for antenna movement in state transitions.

### *Author contribution statement*

AVR designed the project and co-wrote the manuscript. AMY performed the experiments and co-wrote the manuscript. MKZ provided the plant seeds and contributed to the writing.

## Electronic supplementary material

Below is the link to the electronic supplementary material.

Supplementary material 1 (DOCX 758 kb)

## Abbreviations

Cybrid: Cytoplasmic hybrid
*Nt*(*Hn*) cybrid: A cybrid plant combining the nucleus of *Nictotiana tabacum* (tobacco) and the reconstructed cytoplasm of *Hyoscyamus niger* (henbane)
LHCII: Light-harvesting complex of photosystem II
PSII: Photosystem II
RCII: Reaction centre complex of PSII
CP24,26,29: Minor PSII antenna complexes
D1: One of the RCII polypeptides
PAM: Pulse-amplitude-modulated fluorimetry
SP: Saturating pulse
*F*_m_ and *F*_o_: Chlorophyll fluorescence levels when RCII are all closed or all open, respectively
*F*_s_: Steady-state fluorescence levels in the light
*F*_v_: Variable chlorophyll fluorescence (*F*_v_ = *F*_m_ − *F*_o_)
IB: State transitions imbalance parameter (IB = (*F*_s_I′ − *F*_s_I)/*F*_o_)
qS: State transitions *F*_s_ parameter (qS = (*F*_s_I′ − *F*_s_II′)/(*F*_s_I′ − *F*_s_II))
qT: State transition *F*_m_′ parameter (qT = (*F*_m_I* − F*_m_II)/*F*_m_I)
NPQ: Non-photochemical chlorophyll fluorescence quenching
qP: Photochemical chlorophyll fluorescence quenching
SD: Standard deviation
IEF: Isoelectric focusing
