# Supplementary material for: Absence of photosynthetic state transitions in alien chloroplasts
Source: Planta. 2019 May 27;250(2):589–601. doi: 10.1007/s00425-019-03187-2 (PMC6602992; doi:10.1007/s00425-019-03187-2)
Supplement: Supplementary file 1 — Supplementary material 1 (DOCX 758 kb) [file 425_2019_3187_MOESM1_ESM.docx]

**Yeates and Ruban, Supplementary Table 1.**

**Supplementary Table 1**. Identification and N-terminal analysis of *Nt*(*Hn*) LHC proteins by mass spectrometry. The top five hits from the database search are shown for each SDS-PAGE band, 1 and 2 in tobacco and A-F in *Nt*(*Hn*). The sequence similarity of the aligned peptides to the mature protein sequence is given as a percentage. We also indicate whether the alignment was to tobacco (*N.t*) or *N.* *sylvestris* (*N.s*.).


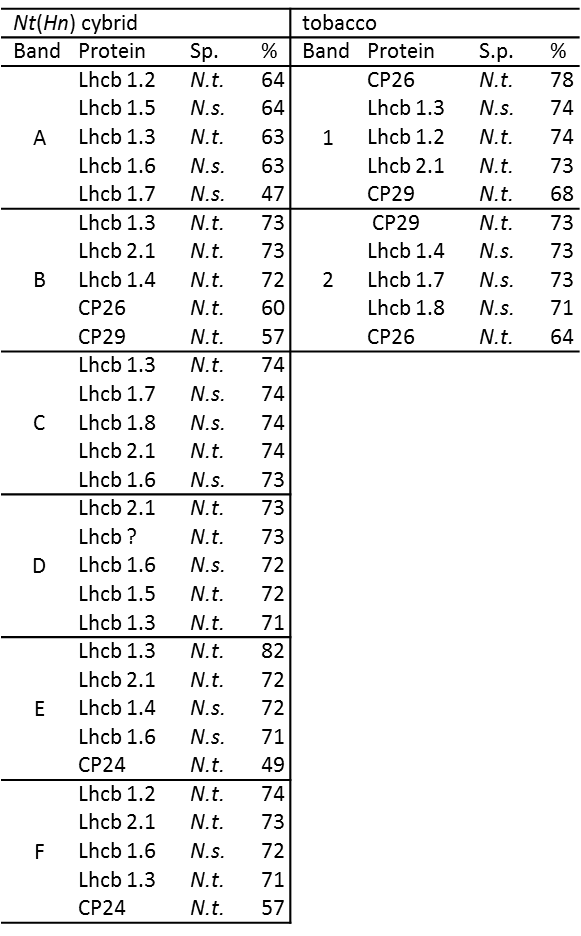


**Yeates and Ruban, Supplementary Figure 1.**


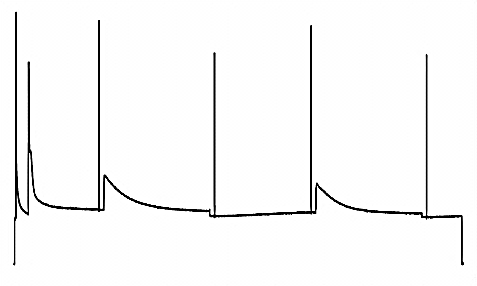


FR

F_m_I’

F_s_I

F_s_I’

F_o_

F_s_II

F_s_II’

F_m_II’

*qT*

*qS*

*IB*

**ST II**

**ST I**

F_m_

**Fluorescence intensity**

**ST I**

**ST II**

on

off

on

on

off

**time**

I

II

**Supplemental Figure 1.** A typical fluorescence trace revealing state transitions during a PAM measurement is labelled with the various measured and calculated parameters used for the quantification of state transition. F_o_, minimal fluorescence; F_m_, maximal fluorescence in a dark adapted sample; F_m_I’ and F_m_II’ maximal fluorescence in a light adapted sample in State I and State II respectively; F_s_I and F_s_I’ are F_s_ levels in State I just before and after the removal of far red light (FR) respectively; F_s_II’ and FsII are F_s_ levels in State II, just before and after the addition of FR; IB, a measure of energy imbalance upon removal of far red light defined as (F_s_I' – F_s_I)/F_o_; qT, defined as (F_­m_I - F_m_II )/F_m_I, reflects differences in the PSII cross-section between State I and State II; qS, defined as (F_s_I' - F_s_II')/(F_s_I' – F_s_II) indicates how fully state transitions have managed to rebalance energy flow between the photosystems. Open triangles at the bottom of the figure indicate when a saturating light pulse was applied. Two closed triangles mark the moments at which the leaf was removed and the tissue excised for 77 K fluorescence measurements in STI (I) and STII (II).

*Nt*(*Hn*)

Tobacco

Henbane

Wavelength, nm

Fluorescence intensity, a.u.

**Supplemental Figure 2.** 77K fluorescence emission spectra of leaf homogenates from tobacco, henbane and *Nt*(*Hn*) cybrid plants recorded in the states 1 (in blue) and 2 (in red). Spectra are normalised to the 685 nm band. The spectra shown here are single measurements. The reproducibility of the experiment was affected by the reabsorption artefacts visible from the red shift of the PSII 685 nm band and altered F685/F735 bands’ ratio. All the spectra on the figure were not distorted by the reabsorption since all the PSII fluorescence maxima were close to 685 nm.


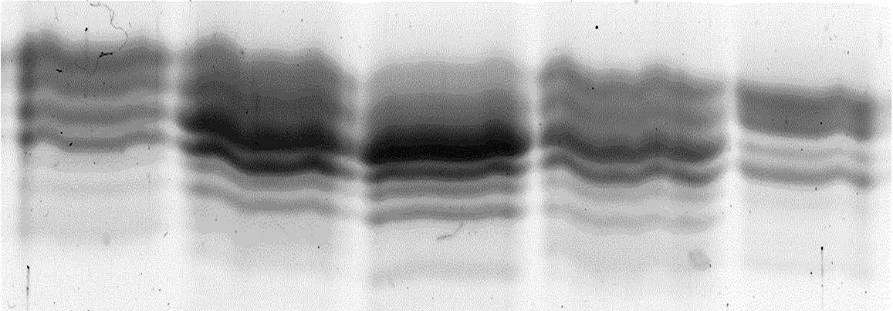


B

A

1

2

C

D

E

F

*T*

*I* *II III IV*

**pI**  4.1 - 3.8 3.8 - 3.5 3. 5 - 3.2 3.2 - 2.9 4.2 - 3.3

**Supplemental Figure 3.** LHCII polypeptides separated by SDS-PAGE. To prepare samples for proteomic investigation, LHCs from tobacco and *Nt*(*Hn*) were first isolated by IEF. The main green band of *Nt*(*Hn*) was divided into four 5 mm strips (I-IV), collected and eluted. The four *Nt*(*Hn*) fractions and the tobacco LHCs (collected together from the broad IEF-band) were then subjected to SDS-PAGE followed by Coomassie staining. *Nt*(*Hn*) bands (A-F), and tobacco bands (1-2), indicated in the figure, were excised from the gel and sent for proteomic analysis.


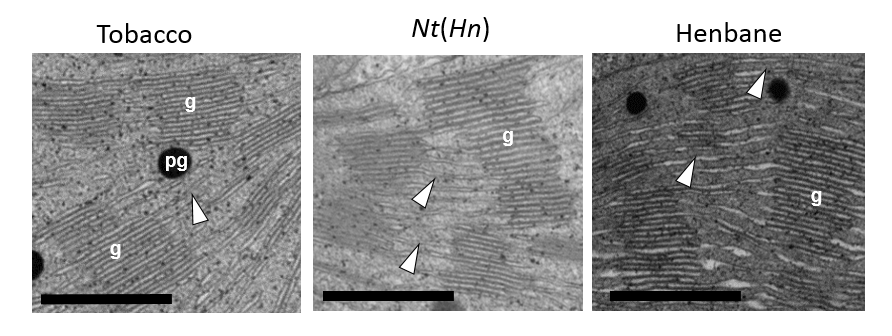


**Supplemental Figure 4.** TEM micrographs of leaf tissue thylakoid membranes in sectioned leaf tissue. Grana (g), stroma lamellae (white arrowheads)

and plastoglobulin (pg) are indicated. Scale bars represent 500 nm.
